# Unusual cause of appendicitis. A case report of acute appendicitis caused by needle ingestion

**DOI:** 10.1016/j.ijscr.2020.05.094

**Published:** 2020-06-12

**Authors:** Francisco Tustumi, Guilherme Garcia Hudari, Natalia Rebeque Modolo, André Luiz Gioia Morrell, Antônio Afonso de Miranda Neto, André Roncon Dias

**Affiliations:** a FMABC, Santo André, SP, Brazil; b FMUSP, São Paulo, SP, Brazil; c Instituto Morrell, Brazil

**Keywords:** Appendicitis, Appendectomy, Ingested foreign body

## Abstract

**Introduction:**

Appendicitis is an acute inflammatory process. The purpose of this report is to describe an unusual case of acute appendicitis in adult caused by accidental ingestion of a needle, and treated with appendectomy.

**Presentation of case:**

A 64 year-old white woman, seamstress, was admitted with abdominal pain complaints. Plain radiograph and CT scan showed metal density suggesting a foreign body in the lower right abdomen. A diagnostic laparoscopic procedure identified a perforated appendicitis due to a needle perforation. Laparoscopic appendicectomy and foreign body removal were performed.

**Discussion:**

Acute appendicitis due foreign body ingestion is extremely rare. Laparoscopy was the chosen access once it was more suitable for inventory of the gastrointestinal tract and help avoiding harm of the medical team during hand maneuvers of open access surgery.

**Conclusion:**

Foreign body ingestion should be remembered as an unusual differential diagnosis of acute appendicitis.

## Highlights

• Appendicitis is defined as an acute and purulent inflammatory process.
• It is the most common cause of surgical acute abdomen.
• This report describes an unusual case of acute appendicitis caused by accidental needle ingestion treated with appendectomy.

## 1 Introduction

Acute appendicitis is the most common surgical urgency [1]. The main causes of acute appendicitis are due to luminal obstruction (by faecolith, lymphoid hyperplasia, or rarely by an neoplasm) [2].

Acute appendicitis associated to foreign body ingestion is extremely rare. Most of the ingested foreign bodies pass through the whole gastrointestinal system spontaneously without causing obstruction or perforation [3]. Perforations or obstruction occur more often in certain sites, such as the ileoceacal valve and the sigmoid [4]. Few case reports of foreign body ingestion inducing acute appendicitis are described

The aim of this study is to report an unusual cause of adult acute appendicitis: ingestion of a needle. This manuscript has been reported in line with the SCARE criteria [5].

## 2 Case report

A 64 year-old white woman, seamstress, was admitted in a urgent care unit complaining of 24 h of right lower quadrant abdominal pain associated with constipation, anorexia, mild fever, chills and malaise. No significant past medical history was mentioned.

Vital signs assessment revealed normal blood pressure, normal heartbeat frequency with 68 beats/min, non-elevated respiratory rate with 16 breaths/min of respiratory rate and 36 °C body temperature. No relevant drug, family, or psychosocial history. Patient’s physical examination revealed mild tenderness during palpation of right lower part of the abdomen, however not suggesting clinical peritonitis.

Laboratory results showed Hemoglobin: 14.5 g/dL, Hematocrit: 44.7%, Leukocytes: 11.5 × 10^3^/μL, Platelets: 254 × 10^3^/μL. Amylase, lipase, and liver enzymes were within normal limits.

**Fig. 1:**
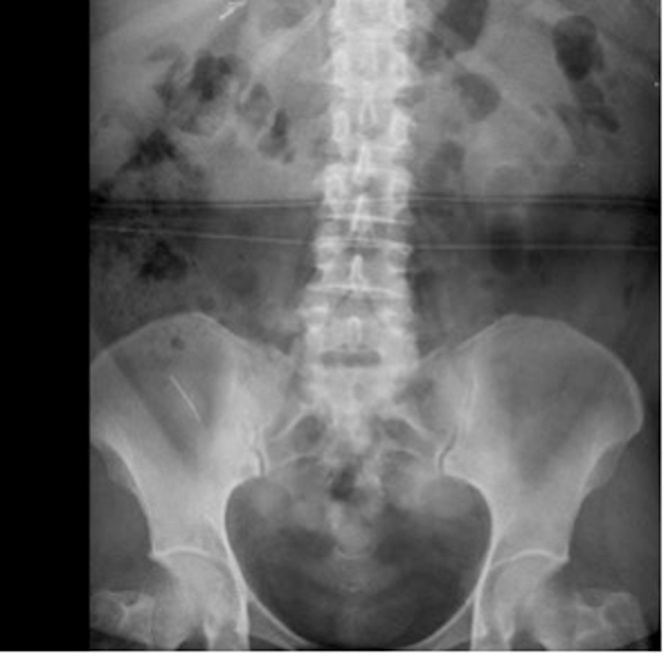
Radiograph showing metal density foreign body in the lower right quadrant.

**Fig. 2:**
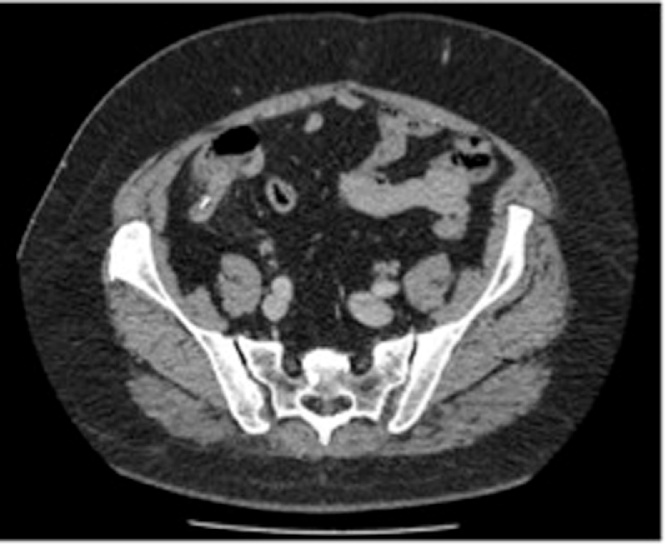
CT showing foreign body within the appendix lumen.

Plain radiograph revealed a metal density foreign body in the right lower quadrant (see Fig. 1). Computed tomography (CT) showed foreign body within the appendix lumen (see Fig. 2).

**Fig. 3:**
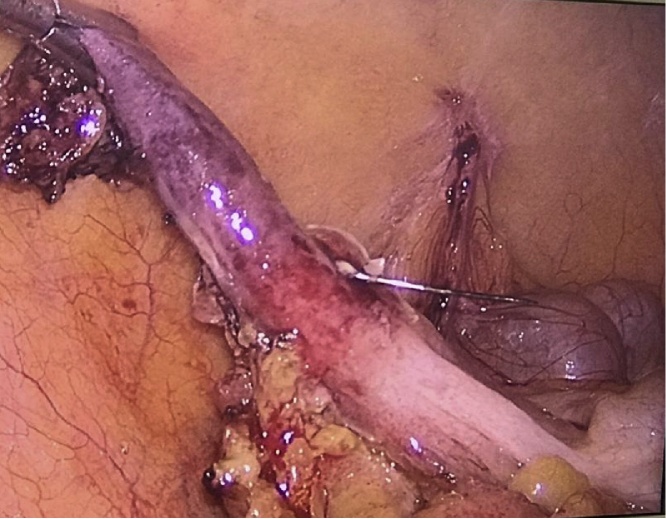
The needle transfixing a hyperemic vermiform appendage.

**Fig. 4:**
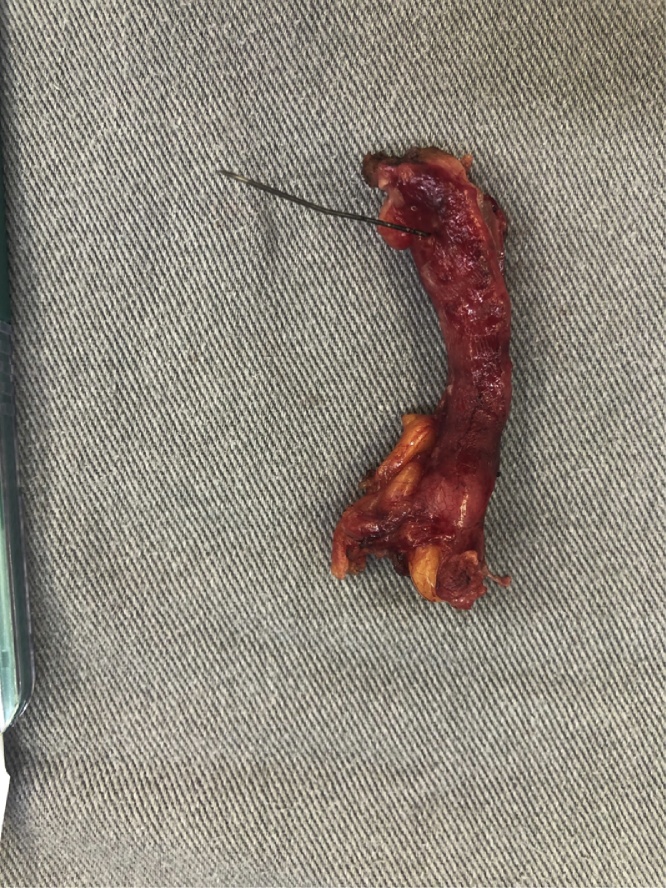
Surgical specimen: the needle transfixing a hyperemic vermiform appendage.

**Fig. 5:**
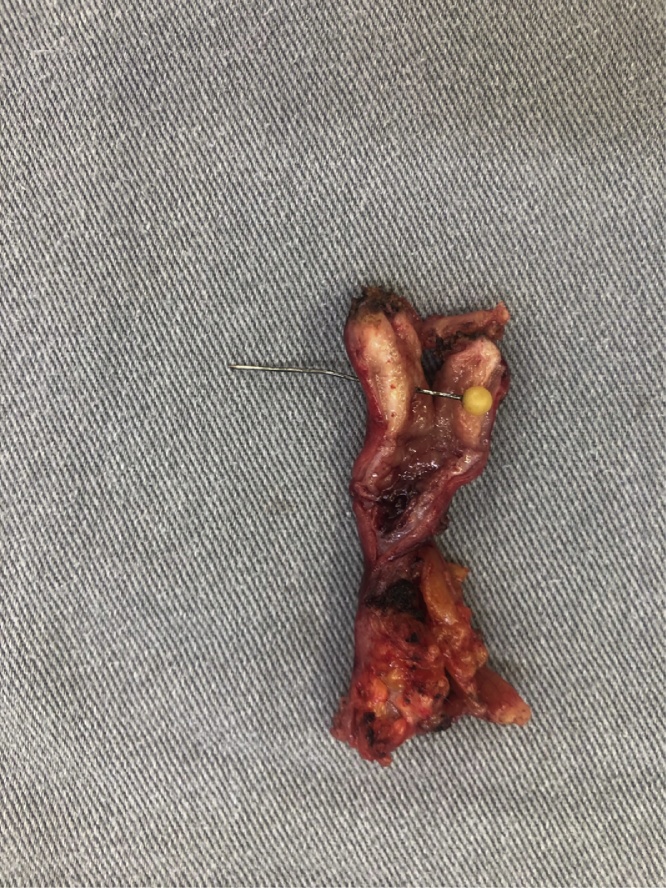
Surgical specimen: vermiform appendage lumen with the needle.

**Fig. 6:**
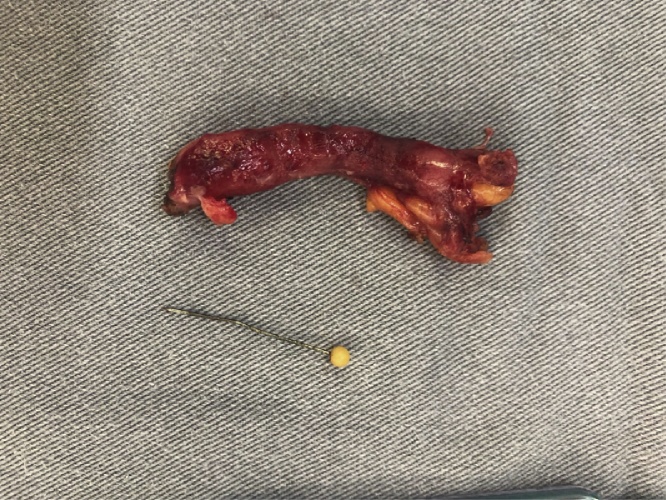
Surgical specimen: vermiform appendage and foreign body (needle).

A diagnostic laparoscopy was indicated and performed by a gastrointestinal surgeon, after a carefully clinical and imaging analysis. After laparoscopic optical introduction, a vermiform appendix with signs of inflammation related to the needle perforation was observed. An appendicectomy was then performed with carefully maneuvers to avoid injury to adjacent structures (see Fig. 3, Fig. 4, Fig. 5, Fig. 6). Other intestinal segments and intra-abdominal organs did not present any sign of injury. Postoperative period was uneventful and patient was discharged after 2 days. Patient was free of symptoms.

Histopathological analysis of specimen revealed ulcero-flegmonous acute appendicitis, with acute fibrine leukocyte peritonitis.  

## 3 Discussion

The present study reports an adult patient with vermiform appendix perforation cause by needle ingestion. Other studies have reported acute appendicitis due to foreign bodies. The cause of foreign body ingestion may be accidental or intentional. The accidental ingestion accounts for most cases and is more commonly seen in the pediatric population or old patients with dementia or other mental disorders. Intentional ingestion is more common in adults as suicidal attempts or psychiatric disorders behavior. In the present study the patient was a seamstress, used to hold needles in her mouth, and did not even notice the foreign body was ingested.

The acute appendicitis due to foreign body ingestion may also be classified as obstruction or perforation. Antonacci et al. [6] reported a blunt material associated to vermiform appendix lumen obstruction. Usually, blunt material is more likely to stay long periods before causing the initial symptoms [6]. Other studies also reported appendiceal obstruction by foreign bodies, and some of them, appendicectomies were performed in a prophylactic manner, once the signs of acute appendicitis were not set yet [[7], [8], [9], [10], [11], [12]].

Appendiceal perforation due to foreign body may present as more acute symptoms than the luminal obstruction of the vermiform appendix. The foreign bodies are usually sharp, stiff, thin, and pointed, such as fishbone or toothpick. Few reports have described cases like these [[13], [14], [15], [16], [17]].

As previous mentioned, a diagnostic laparoscopy was opted for the initial surgical approach. The advantages of a minimally invasive procedure are several, assembling less abdominal wall trauma; lower surgical site infection rates and more suitable for a whole abdominal cavity inspection and identifying possible lesions in gastrointestinal tract [18]. Not less important, a laparoscopy approach would accurately identify the location of the needle, avoiding harm of the medical team during hand maneuvers in an open access surgery.

## 4 Conclusion

Foreign body ingestion should be remembered as an unusual differential diagnosis of acute appendicitis. Laparoscopy should be strongly considered once it allows a more suitable inventory of the gastrointestinal tract and may help avoiding harm of the medical team during hand maneuvers of the open access surgery.

## Declaration of Competing Interest

The authors declare no conflict of interest.

## Sources of funding

The authors received no specific funding for this work.

## Ethical approval

Ethical approval exemption was given for this study.

## Consent

Written informed consent was obtained from the patient for publication of this case report and accompanying images. A copy of the written consent is available for review by the Editor-in-Chief of this journal on request.

## Registration of research studies

Not applicable.

## Guarantor

Francisco Tustumi.

## Provenance and peer review

Editorially reviewed, not externally peer-reviewed.

## CRediT authorship contribution statement

**Francisco Tustumi:** Methodology. **Guilherme Garcia Hudari:** Conceptualization. **Natalia Rebeque Modolo:** Writing - original draft. **André Luiz Gioia Morrell:** Writing - review & editing, Formal analysis, Investigation. **Antônio Afonso de Miranda Neto:** . **André Roncon Dias:** Validation, Supervision.
